# Evaluation of Plasma Soluble Cd40 Ligand Levels in Children with Familial Mediterranean Fever and Its Relationship with Carotid Intima–Media Thickness

**DOI:** 10.3390/diagnostics15091077

**Published:** 2025-04-24

**Authors:** Sukriye Ozde, Cansu Mehdizade, Ozel Mehmet Ali, Cem Ozde, Osman Kayapinar, Muferret Erguven

**Affiliations:** 1 Department of General Pediatric, Faculty of Medicine, Duzce University, 81620 Duzce, Turkey; sukriyeozde@gmail.com (S.O.); cansutamturk3@gmail.com (C.M.); 2 Department of Radiology, Faculty of Medicine, Duzce University, 81620 Duzce, Turkey; drmaliozel@gmail.com; 3 Department of Cardiology, Faculty of Medicine, Duzce University, 81620 Duzce, Turkey; drcemozde@gmail.com; 4 Department of Pediatric Rheumatology, Faculty of Medicine, Duzce University, 81620 Duzce, Turkey; muferete@yahoo.com

**Keywords:** Familial Mediterranean fever, soluble CD40 ligand, carotid intima–media thickness

## Abstract

**Background/Objectives**: It has been suggested that chronic inflammatory diseases may be associated with an increased risk of cardiovascular disease. In this study, we investigated plasma soluble CD40 ligand (sCD40L) levels and their association with carotid intima–media thickness (cIMT) in children with Familial Mediterranean fever (FMF). **Methods**: The study was designed as a prospective cross-sectional study. The study included 68 asymptomatic children aged 5–18 years with FMF, diagnosed according to Tel Hashomer criteria, who were followed up regularly for at least one year, receiving regular colchicine treatment and not in an acute exacerbation period, along with 65 healthy children with similar demographic characteristics. cIMT was assessed by ultrasound and plasma sCD40L levels were measured by sandwich ELISA in all children. **Results**: Erythrocyte sedimentation rate, high-sensitivity C-reactive protein and serum amyloid A levels were not significantly different between patients and controls. However, sCD40L (*p* = 0.004) and fibrinogen (*p* = 0.011) levels were significantly higher in the FMF group compared to the control group. No significant difference was found between the patient and control groups in terms of carotid intima–media thickness (*p* = 0.517). Multivariate logistic regression was performed to assess the independent associations of fibrinogen and sCD40L with FMF. The results of this analysis indicated that sCD40L, but not fibrinogen, demonstrated a significant association with FMF (odds ratio [OR]: 1.003, 95% confidence interval [CI]: 1.001–1.006, *p* = 0.011). To determine the diagnostic performance of sCD40L, a receiver operating characteristic (ROC) curve was generated. This analysis demonstrated that sCD40L levels exceeding 100 pg/mL were predictive of FMF, yielding a sensitivity of 70.6% and a specificity of 62.3%. The positive predictive value and negative predictive value were 55.4% and 64.3%, respectively. The area under the curve for sCD40L was 0.644 (95% CI: 0.549–0.738, *p* = 0.004), signifying a statistically significant predictive capacity. Plasma sCD40L levels were significantly higher in FMF children with the M694V mutation (*p* = 0.013). **Conclusions**: The results of this study suggest that the high plasma sCD40L levels found in children with FMF may be related to the inflammatory activation of the disease rather than to atherosclerosis.

## 1. Introduction

Familial Mediterranean fever (FMF) is the most common autosomal recessive autoinflammatory disease characterised by recurrent fever and inflammation of the serosa. Although FMF is mainly found in people of Mediterranean origin, it has spread worldwide due to large population movements in the twentieth century. The estimated prevalence of FMF is 1/1073, and the carrier rate is approximately 1/5. FMF is characterised by symptoms such as arthralgia, chest pain, erysipelas-like erythema, and fever, leading to serositis, synovitis, pericarditis, pleuritis, and peritonitis. Subclinical inflammation persists even in the periods between acute exacerbations of FMF when patients remain asymptomatic [1].

CD40 and CD40 ligand are membrane glycoproteins of the tumour necrosis factor (TNF-α) family. The interaction pathway between CD40 and CD40L is involved in numerous inflammatory processes by initiating cellular and humoral immunity [2]. Although soluble CD40 ligand (sCD40L) is synthesised by a variety of cells, including activated T cells, macrophages, endothelial cells, and smooth muscle cells, the major source of sCD40L in plasma is activated platelets [2,3]. sCD40L promotes the activation of immune-inflammatory cells, the secretion of inflammatory adhesion molecules, and the migration of leukocytes to the site of inflammation. It also stimulates the tissue factor and triggers the coagulation cascade. Due to its multiple pro-inflammatory effects, sCD40L has been implicated in the initiation and progression of endothelial damage and complications, such as atherosclerotic plaque formation and atherothrombosis [3,4]. The fact that sCD40L is both a pro-inflammatory and a pro-atherosclerotic marker has allowed the relationship between some chronic inflammatory diseases and atherogenicity to be investigated. In chronic inflammatory diseases, such as rheumatoid arthritis, systemic lupus erythematosus, Behçet’s disease, Kawasaki’s disease, and Sjögren’s syndrome, sCD40L levels have been studied and it has been suggested that they may be associated with the risk of cardiovascular disease [5,6,7,8,9].

The development of atherosclerosis commences in a preclinical phase, often decades prior to the emergence of overt cardiovascular disease (CVD) [10]. Employing ultrasound to evaluate subclinical atherosclerosis enhances the precision of CVD risk stratification [11]. Carotid intima–media thickness (cIMT) measurement via ultrasound offers a non-invasive approach to risk evaluation, serving as an indirect indicator of systemic arterial atherosclerosis [12]. Furthermore, it facilitates the evaluation of early-stage, subclinical atherosclerotic alterations within the arterial wall, and enables the monitoring of these changes longitudinally [13]. Contemporary clinical practice guidelines advocate for cIMT measurement in children and adolescents, utilising this non-invasive technique to assess atherosclerotic burden for the purpose of predicting a ten-year cardiovascular risk [10].

In this study, we evaluated plasma sCD40L levels in asymptomatic children with FMF who were not in the acute exacerbation phase and investigated their association with cIMT.

## 2. Materials and Methods

### 2.1. Study Population

This investigation employed a prospective, cross-sectional design. Participants in the patient cohort were recruited from individuals receiving care at the Pediatric Rheumatology Department of Düzce University. Specifically, the patient group comprised asymptomatic children and adolescents, aged 5 to 18 years, who had received a clinical diagnosis of Familial Mediterranean fever (FMF) based on either the Tel-Hashomer or Yalçınkaya criteria. Genetic confirmation of the diagnosis was established via MEFV gene mutation analysis. All patients had been under consistent follow-up for a minimum of one year, maintained on colchicine therapy, and were free from acute exacerbations at the time of enrollment [14]. The PRAS and ISSF scores are summarised in Table 1 and Table 2, respectively [15,16]. The study evaluated responses to colchicine as complete or partial based on MEFV gene mutation characteristics, including subclinical inflammation. The PRINTO criteria were used to classify patients as homozygous, combined heterozygous, and heterozygous mutations, based on the presence or absence of pathogenic MEFV mutations, including the M694V mutation (M694V/M694V, M694V/-). A partial response to treatment was defined as the need for additional medication to reduce the frequency, severity, or duration of attacks [17]. Subclinical inflammation was defined as high levels of sensitive C-reactive protein, erythrocyte sedimentation rate, plasma fibrinogen, and serum amyloid A protein above the normal reference range that could not be explained by other causes during asymptomatic periods, excluding acute exacerbation periods of FMF. Patients with FMF who were overweight, obese, malnourished, had developed amyloidosis, were resistant to colchicine, or were using biologic agents were excluded from the study. Patients with a history of acute or chronic infection, Behçet’s disease, juvenile idiopathic arthritis, collagen diseases, systemic lupus erythematosus, vasculitis, epilepsy, migraine, type 1 and type 2 diabetes mellitus, acute or chronic renal disease, hypertension, dyslipidaemia, sleep apnoea syndrome, congenital or acquired heart disease, smoking and alcohol use were also excluded. The control group consisted of completely healthy children admitted to the Healthy Child Outpatient Clinic of the Department of General Paediatrics. In this context, the patient group consisted of 68 patients and the control group of 65 patients. All subjects (patients and controls) were enrolled in the study between August 2022 and February 2024. Written informed consent was obtained from the parents of all patients included in the study. The demographic, anthropometric, and clinical characteristics of all patients enrolled in the study were recorded, and the patients then underwent a detailed physical examination. The study protocol was approved by the Institutional Ethics Committee of Düzce University Faculty of Medicine and was conducted in accordance with the Declaration of Helsinki (Decision no. 2022/134 dated 4 July 2022).

### 2.2. Measurement of Soluble CD40 Ligand

Following an overnight fast, three millilitres of venous blood were obtained from the antecubital fossa during the morning hours. The collected blood was promptly transferred into evacuated tubes containing 3.8% sodium citrate (BD Biosciences, San Jose, CA, USA) and subsequently subjected to centrifugation at 2000 rpm for 20 min at a temperature of 4 °C. The resultant plasma was separated into aliquots and stored at −80 °C until analysis. Plasma sCD40 ligand (sCD40L) concentrations were quantified utilising a commercially available enzyme-linked immunosorbent assay (ELISA) kit (eBioscience, San Diego, CA, USA). This assay exhibits a lower limit of detection of 0.06 ng/mL, with inter-assay and intra-assay coefficients of variation below 6.0% and 5.5%, respectively. The assay demonstrates high specificity for human sCD40L, with the manufacturer reporting cross-reactivity with related molecules of less than 0.5%. The established reference interval for sCD40L levels is 0.16 to 10 ng/mL.

### 2.3. Laboratory Measurements and Formulations

The blood samples were collected simultaneously with the sCD40L sampling. Biochemical blood tests were analysed using the Cobas e602 analyser (Roche, Berlin, Germany). Circulating high-sensitivity C-reactive protein (hsCRP) was quantified through nephelometry, employing the Cobas c702 analyzer (Roche, Berlin, Germany). Fasting glucose levels were assessed via the hexokinase enzymatic assay. Total cholesterol was determined using the enzymatic colorimetric method. High-density lipoprotein (HDL) cholesterol was measured utilising an accelerator selective detergent methodology. Triglyceride levels were ascertained through a glycerol-3-phosphate oxidase enzymatic reaction. Low-density lipoprotein (LDL) cholesterol was calculated using the Friedewald equation. A complete blood count was performed on the LH500 Automated Hematology Analyzer (Beckman Coulter, Budapest, Hungary). Plasma fibrinogen levels were determined by the Clauss clotting assay, utilising the Diagon Coag XL system (Budapest, Hungary). Erythrocyte sedimentation time and ferritin levels were also measured. The atherogenic index of plasma (AIP) was calculated by dividing the values of triglycerides (TG) by the values of high-density lipoprotein (HDL) and taking the logarithm to base 10 [log (TG/HDL)]. The results were classified as low risk (<0.11), intermediate risk (0.11–0.21), and high risk (>0.21) [18].

### 2.4. MEFV Gene Mutation Analysis

Peripheral blood samples obtained from the patients were analysed for 22 mutations in the MEVF gene using the Real-Time PCR method. DNA was isolated from peripheral blood using the SNPure^®^ Blood DNA and MN NucleoSpin^®^ Blood kit (Budapest, Hungary), and mutations were detected according to company protocols. The kit treated 200 µL of blood sample with wash buffer 1 (buffer AW1), proteinase K, and wash buffer 2 (buffer AW2) in column tubes at room temperature. The final step involved dilution with sterile water. The concentration was measured, and mutations were detected using a commercially available SNP Biotechnology kit following the company’s protocols. The kit can detect 99.2–99.5% of MEFV mutations observed in the United States and many other countries. It consists of sequence-specific oligonucleotides and ready-to-use reagents specially developed for 5’nuclease PCR used in SNP analysis.

### 2.5. Measurement of Carotid Intima–Media Thickness

Common carotid intima–media thickness (cIMT) was evaluated in all participants utilising a GE Logiq S8 ultrasound system (General Electric Healthcare, Chicago, IL, USA; 2018 model) equipped with a 6–10 MHz linear transducer. Electrocardiographic gating was employed to synchronise imaging with diastole. A radiologist, blinded to patient clinical data, conducted all ultrasound examinations. Studies were performed following an overnight fast, in a temperature-controlled, quiet environment. With participants in a supine position and neck extended, the distal 10 mm of both left and right common carotid arteries, just proximal to the carotid bulb, were visualised at a fixed magnification. Images were acquired only when both anterior and posterior vessel wall borders were clearly defined, ensuring perpendicular beam incidence. The highest quality images from each carotid artery were stored. Subsequent to data collection, a single radiologist performed offline analysis of all images. Intimal thickness was manually measured at three distinct locations within each carotid artery, and the average measurement was computed. The final cIMT value was determined by averaging the mean measurements obtained from the right and left carotid arteries [10]. Intra-observer reliability was assessed by repeating measurements in a randomly selected subset of 15 participants under identical conditions. Inter-observer reliability was evaluated by a second observer performing measurements from recorded ultrasound videos. The reproducibility of cIMT measurements was quantified by calculating the coefficient of variation (CV) between repeated measurements. The CV was calculated by dividing the standard deviation of the differences between repeated measurements by the mean of the repeated measurements and expressed as a percentage. Intra-observer CV was determined to be below 5%.

### 2.6. Statistical Analysis

The distribution of variables was assessed for normality through both visual inspection of histograms and probability plots, as well as analytical evaluation using the Shapiro–Wilk test. For normally distributed variables, descriptive statistics are presented as means and standard deviations. Non-normally distributed data are described using medians, ranges, and frequencies. The Kolmogorov–Smirnov test was employed to further evaluate data distribution. Quantitative data were compared using analysis of variance (ANOVA) with Tukey post hoc testing, or the non-parametric Kruskal–Wallis and Mann–Whitney U tests, as appropriate. Qualitative data were analysed using the chi-squared (χ^2^) test. Statistical significance was established at a *p*-value of less than 0.05. The predictive capacity of sCD40L levels for Abdominal Aortic Aneurysm (AAA) was evaluated through receiver operating characteristic (ROC) curve analysis. Sensitivity and specificity were calculated for any statistically significant cutoff values. The area under the ROC curve was considered significantly predictive at a 5% type I error level. Spearman’s rank correlation coefficient was used to assess correlations between variables. Univariate and multivariate logistic regression analyses were performed to evaluate effect sizes. All statistical analyses were conducted using SPSS version 28.0.

## 3. Results

Demographic and basic clinical data are summarised in Table 3. There were no significant differences in age, BMI, blood pressure, heart rate, and body temperature between the two groups. Comprehensive clinical characteristics of the Familial Mediterranean fever (FMF) patient cohort are provided in Table 4.

The results of the blood biochemistry and complete blood count analyses are outlined in Table 5. Fasting blood glucose, insulin, creatinine, alanine aminotransferase, aspartate aminotransferase, total cholesterol, high-density lipoprotein, low-density lipoprotein, triglycerides, thyroid stimulating hormone, vitamin D, vitamin B12, total white blood cell count, platelet count, and haemoglobin levels were evaluated, and no significant difference was found between the patient and control groups.

As displayed in Table 6, there were no statistically significant differences observed between the patient and control groups regarding carotid intima–media thickness (0.40 ± 0.05 vs. 0.40 ± 0.03; *p* = 0.517) or plasma atherogenic index.

The results of the plasma sCD40L and conventional inflammatory biomarkers are summarised in Table 7. Erythrocyte sedimentation rate, high-sensitivity C-reactive protein, serum amyloid A, and ferritine levels did not differ significantly between patients and controls (all *p* value > 0.05). However, sCD40L (250.7 ± 194.9 vs. 155.5 ± 137.7; *p* = 0.004) and fibrinogen (238.9 ± 75.3 vs. 216.9 ± 54.8; *p* = 0.011) levels were significantly higher in the FMF group compared to the control group.

To assess the independent associations between fibrinogen, sCD40L, and Familial Mediterranean fever (FMF), multivariate logistic regression analysis was conducted. The results, detailed in Table 8, indicated that only sCD40L exhibited a significant association with FMF (Exp(B)/OR: 1.003, 95% CI: 1.001–1.006, *p* = 0.011).

**Figure 1:**
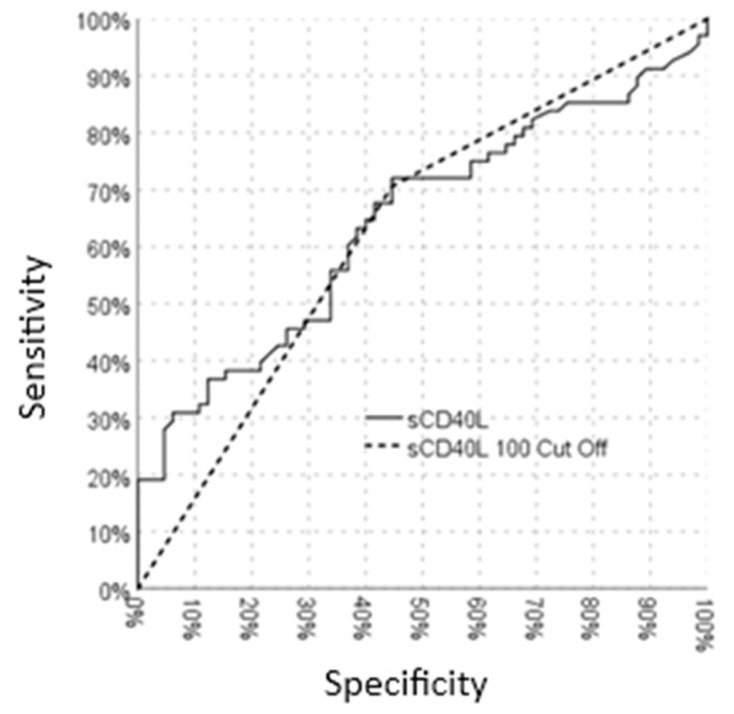
ROC curve analysis.

ROC curve analysis was employed to determine the diagnostic utility of sCD40L in differentiating children with FMF from controls. As depicted in Figure 1, the ROC curve analysis revealed that an sCD40L value exceeding 100 pg/mL demonstrated a statistically significant association with FMF (area under the curve [AUC]: 0.644; 95% CI: 0.549–0.738; *p* = 0.004). At this cutoff point, sCD40L exhibited a sensitivity of 70.6% and a specificity of 62.3%, with a positive predictive value of 55.4% and a negative predictive value of 64.3%.

The association between MEFV mutations and plasma sCD40L levels in children with FMF is summarised in Table 9. Plasma sCD40L levels were significantly higher in FMF patients with the M694V mutation (336.6 ± 220.1 vs. 209.6 ± 169.2, *p* = 0.013).

## 4. Discussion

This study is the first to investigate plasma sCD40L levels and their association with cIMT in children with FMF. The main findings of the study can be summarised as follows: Plasma sCD40L levels were significantly higher in asymptomatic children with FMF who were not in acute exacerbation compared to healthy controls. However, no significant difference in carotid intima–media thickness was observed between children with FMF and healthy controls. Furthermore, no association was identified between plasma sCD40L levels and carotid intima–media thickness. Additionally, plasma sCD40L levels were found to be significantly elevated in children carrying the M694V mutation.

FMF is a chronic inflammatory disease with exacerbations characterised by symptoms such as fever, peritonitis, and pleuritis, self-limiting attacks occur within a few days, interspersed with asymptomatic periods between exacerbations. Although acute exacerbations are clinically suppressed by colchicine prophylaxis, it is known that there is increased inflammatory activity during the asymptomatic periods. The mutant pyrin protein formed as a result of the MEFV gene mutation and the production of pro-inflammatory cytokines such as interleukin-1ß (IL-1ß), IL-6, IL-8, and TNF-α caused by this mutation, as well as the persistence of inflammatory activity in the non-exacerbation period, are referred to as subclinical inflammation [19]. It is thought that chronic inflammation, which persists with circulating inflammatory proteins and activated immune system cells, is responsible for the short- and long-term complications of FMF and the prognosis of the disease. In addition, chronic inflammation may predispose individuals to cardiovascular disease, such as endothelial dysfunction [20,21]. In this context, it has been debated whether chronic inflammatory diseases, such as FMF, may predispose to atherosclerotic cardiovascular disease.

Recent studies have investigated pre-atherosclerotic findings in children with FMF. Ghobrial et al. reported that epicardial adipose tissue thickness, which is known to be associated with atherosclerosis and is an important indicator of visceral adiposity, was significantly increased in children with FMF [22]. Peru et al. found that carotid intima–media thickness, a pre-atherosclerotic lesion, was significantly increased in children with FMF [23]. Bilginer et al. found that cIMT was significantly increased in children with FMF compared to healthy controls and associated this finding with subclinical inflammation in the subjects included in the study [24]. In a separate study, Mahmoud et al. reported that cIMT, a marker of subclinical atherosclerosis, exhibited an increase in children suffering from FMF in comparison to the control group. This finding underscores the pivotal role of chronic inflammation in the etiopathology of atherogenesis [25]. However, Vampertzi et al. found that femoral carotid pulse wave velocity, subendocardial viability, and cIMT were similar to those of healthy controls in a study of children and young adults with FMF [26]. In a different study, Türkuçar et al. reported that serum endocan levels and cIMT in children with FMF were similar to those in healthy controls. They also suggested that good disease control in FMF may have an atheroprotective effect [27]. In the present study, we did not find a significant difference in carotid intima–media thickness in children with FMF compared with healthy controls. The exclusion of children with treatment-resistant FMF, who had more intense chronic inflammatory activity, may be related to this finding. Furthermore, the observed results may be attributed, in part, to the relatively young age demographic of the paediatric participants within this study. Additionally, the relatively brief duration of exposure to FMF in the study subjects may also have contributed to these results.

Smooth muscle cell migration, vascular calcification, increased metalloproteinase activity, extracellular matrix degradation, oxidative stress, elastolysis, and collagen destruction that occur in chronic inflammation may set the stage for the onset and development of atherosclerosis [28]. The CD40-CD40L interaction has been studied in chronic autoimmune and inflammatory diseases because of the cellular and humoral immune responses in which it is involved. Chronic immune–inflammatory diseases, including multiple sclerosis, Graves’ disease, rheumatoid arthritis, systemic lupus erythematosus, Kawasaki disease, and FMF, have been linked to an increased risk of cardiovascular disease [5,6]. Metwalley et al. reported that sCD40L levels were significantly higher in children with newly diagnosed Graves’ disease and this may be related to the pathogenesis of the disease [6]. Urquizu-Padilla et al. found that sCD40L levels were lower in SLE patients during the exacerbation period compared to the remission period and associated this with sCD40L consumption during disease activation [7]. In rheumatoid arthritis, several studies have been performed on the CD40-CD40L pathway and sCD40L was found to be significantly higher in patients with rheumatoid arthritis. Moreover, it is thought that agents that block the CD40/CD40L interaction inhibit disease progression [8]. In a study focusing on the relationship between Behçet’s disease and sCD40L, Fernandez et al. found that sCD40L levels were significantly higher in Behçet’s patients, who are known to have endothelial damage and prothrombotic vasculitis [9]. In this study, plasma sCD40L levels were found to be significantly higher in children with FMF who were not in the acute exacerbation phase and who had no significant increase in traditional inflammatory parameters compared to healthy children. The fact that only plasma sCD40L levels were found to be elevated, without a significant increase in cIMT in the children included in our study, suggests that this finding is more directly related to the chronic inflammatory activity of FMF disease rather than a proatherosclerotic process.

In a study conducted in children, Li et al. reported that CD40/CD40L activation was associated with AST, ALT, total cholesterol, and total platelet levels and that these parameters were factors that could influence sCD40L levels [29]. In this study, no significant difference was found between FMF and healthy children in terms of secondary parameters that may affect plasma sCD40L levels. In this context, the significant increase in plasma sCD40L levels found in children with FMF seems to be directly related to FMF-induced chronic inflammatory activity.

It is known that mutations in exon 10 of the MEFV gene, especially the M694V mutation, cause a more severe clinical course, the need for higher doses of colchicine in treatment, a more complex clinical course, and increased mortality in FMF patients [30,31]. It is thought that increased secretion of pro-inflammatory cytokines in the M694V mutation is associated with increased endothelial damage and cardiovascular disease risk. A recent study by Şahin et al. involving children with FMF who had amyloidosis reported that carotid intima–media thickness was significantly increased in those with the M694V homozygous mutation, compared with those with other homozygous mutations and combined heterozygotes [32]. In the same study, FGF23 and PTX3 levels, which are markers of atherosclerosis and endothelial dysfunction, respectively, were found to be higher in children with the homozygous M694V genotype [32]. In a retrospective study, the rate of myocardial infarction was found to be significantly higher in FMF patients with the M694V mutation [33]. In another study involving a relatively small number of patients, no significant difference was found between FMF patients with and without cardiovascular disease with regard to the M694V mutation [34]. In our present study, the M694V mutation was found in 32.4% of children with FMF. Plasma sCD40L levels were significantly higher in FMF children with the M694V mutation compared to FMF children without the M694V mutation. However, there was no significant difference in cIMT between children with and without the M694V mutation. These findings suggest that plasma sCD40L levels may be associated with disease severity.

The present study is subject to several inherent limitations. Firstly, all participants were undergoing consistent colchicine therapy, thereby precluding the assessment of the impact of patients who were resistant to colchicine on the observed outcomes. Secondly, due to financial constraints, MEFV gene mutation analysis was not conducted on the control group. Given that asymptomatic carriers may exhibit subclinical inflammatory activity [35], the presence of undetected MEFV mutation carriers within the control cohort may have introduced confounding variables. Thirdly, the relatively limited sample size may compromise the generalizability of the findings, and larger-scale studies are warranted to validate these results. Finally, patients with FMF-associated renal amyloidosis were excluded. It is acknowledged that systemic amyloidosis, which can lead to end-stage renal disease, is the most severe complication of FMF [36]. The exclusion of patients with renal amyloidosis may have resulted in an inaccurate cardiovascular risk profile, as individuals with renal amyloidosis may be the FMF subgroup most susceptible to premature cardiovascular disease. In order to validate the current findings, it is essential that future investigations include a larger patient population and encompass all FMF phenotypic presentations.

In conclusion, plasma sCD40L levels were significantly higher in asymptomatic children with FMF. However, carotid intima–media thickness was similar in both groups and did not correlate with plasma sCD40L levels. In addition, plasma sCD40L levels were significantly higher in FMF children with the M694V mutation. The results of this study suggest that the high plasma sCD40L levels found in children with FMF may be related to the inflammatory activation of the disease rather than to atherosclerosis.

## Figures and Tables

**Table 1:** Modified PRAS disease severity scoring.

Starting Age	
	<6 age: 4 points 6–10 age: 3 points >10 age: 2 points
Frequency of Attacks	
	More than 2 attacks per month: 3 points 1–2 attacks per month: 2 points Less than 1 attack per month: 1 point
Articular Involvement	
	Chronic arthritis: 3 points Acute arthritis 2 points
Erysipelas-like Erythema	
	Two points if available
Amyloidosis	
	Three points if available
Colchicine Dose to Control Attacks *	
	Less than the appropriate dose: 0 points Appropriate dose 1 point More than the appropriate dose: 2 points
Mild: 3–5 points, Moderate: 5–9 points, Heavy: ≥10 points	

* Appropriate dose: <5 years 0.5 mg/day, 5–10 years 1 mg/day; >10 years 1.5 mg/day.

**Table 2:** The International Severity Score for FMF (ISSF).

ISSF Criteria	
	Point
Presence of chronic sequelae (amyloidosis, growth retardation, splenomegaly)	1
2. Organ dysfunction (AAA-associated nephrotic proteinuria)	1
3. Organ failure (AAA-related heart, kidney failure)	1
4. a. Attack frequency averaging 1–2 per month b. Attack frequency averaging >2 per month	1 2
5. Elevated acute phase reactants even after the attack	1
6. More than two involvements during the attack (pericarditis, pleuritis, peritonitis, synovitis, testicular involvement, etc.)	1
7. Having more than two different types of attacks during the course of the disease	1
8. At least 3 attacks per year with a duration of more than 72 h	1
9. Leg pain with exercise	1

Mild: ≤2 points, Moderate: 3–5 points, Heavy: ≥6 points.

**Table 3:** Demographic and Basic Clinical Findings.

	FMF Group (n = 68) Mean Value ± SD/(n %) or (n Median Value)	Control Group (n = 65) Mean Value ± SD/(n %) or (n Median Value)	*p* Value
Gender			0.979 ^X2^
Male	26 (38.2%)	25 (38.5%)	
Female	42 (61.8%)	40 (61.5%)	
Age, year	10.9 ± 3.9 (10.0)	10.2 ± 4.2 (9.0)	0.261^m^
BMI, (cm/kg^2^)	16.2 ± 3.3 (16.0)	15.8 ± 2.6 (15.0)	0.692 ^m^
BMI, percentile	33.0 ± 28.1 (22.0)	30.9 ± 24.1 (24.1)	0.836 ^m^
sBP, mmHg	106 ± 7.5 (102.0)	105 ± 4.3 (101.0)	0.108 ^m^
dBP, mmHg	63 ± 5.5 (61.5)	62 ± 5.2 (60.0)	0.455 ^m^
Heart rate, bpm	88.5 ± 3.6 (86.5)	89 ± 3.5 (87.5)	0.801 ^m^
Body Temperature, °C	37.1 ± 0.5 (37.0)	37.2 ± 0.5 (37.0)	0.933 ^m^

^X2^ Ki-kare test, ^m^ Mann–Whitney u test, FMF: Familial Mediterranean fever, BMI: body mass index, sBP: systolic blood pressure, dBP: diastolic blood pressure, bpm: beat per minute.

**Table 4:** Clinical Characteristics of the FMF Patients.

	FMF Group (n = 68) Mean Value ± SD/(n %) or (n Median Value)
Modified Pras Score	
Mild	22 (33.3%)
Intermediate	43 (65.1%)
Severe	3 (4.5%)
ISSF Score	
Mild	37 (56.2%)
Intermediate	26 (39.3%)
Severe	5 (7.5%)
Response to Colchicine Treatment	
Partial response	21 (30.8%)
Full response	47 (69.1%)
Subclinical Inflammation	
[+]	25 (36.7%)
[−]	43 (63.2%)
Age of Diagnosis, Year	7.4 ± 3.2 (6.0)
Total Diagnosis time, Year	3.8 ± 1.6 (3.0)

FMF: Familial Mediterranean fever, I. SSF score: International Severity Scoring System for FMF.

**Table 5:** Blood Biochemistry and Complete Blood Count Results.

	FMF Group (n = 68) Mean Value ± SD or (n Median Value)	Control Group (n = 65) Mean Value ± SD or (n Median Value)	*p* Value
FBG, mg/dL	88.3 ± 6.9 (88.6)	87.4 ± 6.0 (88.0)	0.218 ^m^
Insulin uIU/mL	7.8 ± 4.7 (6.9)	7.0 ± 4.8 (6.2)	0.248 ^m^
sCr, mg/dL	0.49 ± 0.14 0.(49)	0.47 ± 0.14 (0.40)	0.508 ^m^
ALT, IU/L	15.3 ± 5.9 (14.0)	14.1 ± 5.7 (14.0)	0.095 ^m^
AST, IU/L	24.0 ± 7.2 (23.0)	25.4 ± 7.4 (26.0)	0.247 ^m^
Total Cholesterol, mg/dL	147.9 ± 25.9 (148.0)	148.5 ± 28.3 (151)	0.887 ^t^
LDL, mg/dL	82.3 ± 21.1 (80.0)	83.8 ± 26.2 (83.0)	0.771 ^t^
HDL, mg/dL	49.9 ± 11.6 (50.6)	50.2 ± 9.8 (49.0)	0.836 ^t^
Triglyceride, mg/dL	77.4 ± 43.7 (66.5)	82.3 ± 39.0 (72.0)	0.201 ^m^
TSH, mIU/L	2.5 ± 1.2 (2.3)	2.4 ± 1.2 (2.1)	0.249 ^m^
Vitamin D, ng/mL	21.9 ± 8.2 (22.0)	26.0 ± 14.0 (24.0)	0.063 ^m^
Vitamin B12, pg/mL	415.6 ± 229.4 (390.0)	425.2 ± 169.9 (396.0)	0.498 ^m^
WBC, 10^3^/mm^3^	6.5 ± 1.3 (5.9)	6.8 ± 1.1 (6.6)	0.062 ^m^
Neutrophil, 10^3^/mm^3^	2.8 ± 1.0 (2.8)	3.2 ± 1.2 (3.1)	0.052 ^m^
Lymphocyte, 10^3^/mm^3^	2.5 ± 0.7 (2.2)	2.7 ± 1.2 (2.6)	0.058 ^m^
Monocyte, 10^3^/mm^3^	1.1 ± 0.2 (1.1)	1.1 ± 0.3 (1.1)	0.154 ^m^
Basophil, 10^3^/mm^3^	0.1 ± 0.1 (0.1)	0.1 ± 0.1 (0.1)	0.855 ^m^
Eosinophil, 10^3^/mm^3^	0.1 ± 0.1 (0.1)	0.1 ± 0.1 (0.1)	0.766 ^m^
Haemoglobin, g/dL	12.6 ± 1.2 (12.8)	12.6 ± 1.1 (12.6)	0.541 ^m^
Platelet, 10^9^/L	302.8 ± 68.3 (300.0)	296.7 ± 92.3 (274.0)	0.173 ^m^

^m^ Mann–Whitney u test, ^t^ Independent sample *t*-test, FMF: Familial Mediterranean fever, FBG: fasting blood glucose, sCR: serum creatinine, ALT: alanine aminotransferase, AST: Aspartate, Aminotransferase, LDL: low-density lipoprotein cholesterol, HDL: high-density lipoprotein cholesterol, TSH: thyroid stimulating hormone, SD: standard deviation.

**Table 6:** Carotid Intima–Media Thickness and Atherogenic Index of Plasma Values.

	FMF Group (n = 68) Mean Value ± SD/(n %) or (n Median Value)	Control Group (n = 65) Mean Value ± SD/(n %) or (n Median Value)	*p* Value
cIMT, mm	0.40 ± 0.05 (0.40)	0.40 ± 0.03 (0.40)	0.517 ^m^
AIP	−0.21 ± 0.25 (−0.22)	−0.18 ± 0.22 (−0.20)	0.433 ^t^
AIP Risk Group			
Low	61 (%89.7)	56 (%86.2)	0.529 ^X2^
Intermediate	2 (%2.9)	6 (%9.2)	
High	5 (%7.4)	3 (4.6%)	

^m^ Mann–Whitney u test, ^t^ Independent sample *t*-test, ^X2^ Chi-square test, FMF: Familial Mediterranean fever, cIMT: carotid intima–media thickness, AIP: Atherogenic index of plasma, SD: standard deviation.

**Table 7:** Plasma sCD40L and Conventional Inflammation Biomarker Values.

	FMF Group (n = 68) Mean Value ± SD/(n %) or (n Median Value)	Control Group (n = 65) Mean Value ± SD/(n %) or (n Median Value)	*p* Value
sCD40L, pg/mL	250.7 ± 194.9 (207.3)	155.5 ± 137.7 (87.2)	0.004 ^m^
ESR, mm/h	10.7 ± 9.0 (8.0)	10.8 ± 6.6 (9.0)	0.314 ^m^
hs-CRP, mg/dL	0.3 ± 0.9 (0.06)	0.1 ± 0.1 (0.06)	0.456 ^m^
Fibrinojen mg/dL	238.9 ± 75.3 (250.0)	216.9 ± 54.8 (219.0)	0.011 ^m^
SAA, mg/dL	13.4 ± 36.1 (5.1)	4.1 ± 4.1 (3.8)	0.070 ^m^
Ferritin, ng/mL	31.0 ± 19.2 (26.0)	34.6 ± 24.6 (32.0)	0.312 ^m^

^m^ Mann–Whitney u test, sCD40L: soluble CD40 ligand, ESR: erythrocyte sedimentation rate, hs-CRP: high sensitive C-reactive protein, SAA: Serum amyloid A, SD: standard deviation.

**Table 8:** Logistic Regression Analysis.

	Univariate Model			Multivariate Model		
	Odds Ratio	%95 Confidence Interval	*p* Value	Odds Ratio	%95 Confidence Interval	*p* Value
sCD40L	1.003	1.001–1.006	0.002	1.00.3	1.001–1.006	0.011
Fibrinogen	1.005	1.000–1.011	0.041			

sCD40L: soluble CD40 ligand.

**Table 9:** Relationship between MEFV mutations and plasma soluble CD40L level in FMF group.

	sCD40L, pg/mL Mean Value ± SD	*p* Value
M694V mutation		0.013
[+], (n = 22)	336.6 ± 220.1	
[−], (n = 46)	209.6 ± 169.2	
Homozygous, (n = 16)	282.9 ± 228.1	0.223
Combined heterozygote, (n = 31)	276.2 ± 228.1	
Heterozygous, (n = 21)	192.8 ± 182.7	
Pathogenic in PRINTO		0.357
[+], (n = 23)	283.9 ± 221.1	
[−], (n = 45)	233.7 ± 180.3	

## Data Availability

The data used to support the findings of this study are available from the corresponding author upon request.

## Abbreviations

FMF: Familial Mediterranean fever; sCD40L, soluble CD40 ligand; cIMT, carotid intima–media thickness; TNF-α, tumour necrosis factor; CVD, cardiovascular disease; LDL, Low-density lipoprotein; AIP; atherogenic index of plasma; HDL, high-density lipoprotein; TG, triglyceride; ROC, receiver operating characteristic; IL, interleukin.
